# Design and Preliminary Testing of the CardioCare System in Health Checkup Centers: Implementation Report

**DOI:** 10.2196/78942

**Published:** 2026-07-13

**Authors:** Yan Wang, Jingyi Xiao, Xiaoying He, Lei Su, Jiao Wang, Hua Hong, Lin Xu

**Affiliations:** 1 Centre of Health Management, The First Affiliated Hospital, Sun Yat-sen University, Guangzhou, China; 2 Department of Geriatrics, The First Affiliated Hospital, Sun Yat-Sen University, Guangzhou, China; 3 School of Public Health, Sun Yat-sen University, Rm 103, School of Public Health Building, Sun Yat-sen University (North Camp.), #74, Zhongshan 2nd Road, Yuexiu District, Guangzhou, 510080, China, 86 13602477650; 4 School of Public Health, University of Hong Kong, Hong Kong, China (Hong Kong); 5 Department of Applied Health Sciences, University of Birmingham, Birmingham, United Kingdom

**Keywords:** cardiovascular disease prevention, health checkup system, digital health, risk stratification, cardiovascular risk management

## Abstract

**Background:**

Health checkup programs in China reach millions of older adults for cardiovascular risk screening, but few effective mechanisms exist to ensure high-risk individuals receive follow-up preventive care.

**Objective:**

We developed the CardioCare system, a digital tool integrating a 10-year cardiovascular disease (CVD) risk prediction model with personalized health management features, to address this gap.

**Methods:**

The CardioCare system was used in a hospital-based health checkup center. The system incorporates an established 10-year CVD risk model to estimate risk. We conducted preliminary usability testing at a health checkup center in Guangzhou. Older adults identified as high risk (≥10% 10-year CVD risk) were given personalized feedback and invited to a cardiovascular risk management clinic.

**Implementation (Results):**

The CardioCare system was successfully implemented, automatically stratifying CVD risk for each checkup attendee and generating patient-specific recommendations. The CVD risk model integration functioned without major technical issues, although minor performance delays were identified and resolved, and physicians and nurses reported that the tool was user-friendly and fit smoothly into the clinic workflow. However, patient engagement was low: of the 2069 high-risk individuals invited for follow-up care, 181 (8.7%) attended at least 1 in-person visit at the clinic. Feedback from the patients who engaged was positive regarding the clarity of risk information and advice, but the low response rate indicated significant barriers to uptake.

**Conclusions:**

This study demonstrated the technical feasibility of integrating a digital CVD risk assessment and management system into a health checkup setting. The system was successfully embedded into the clinical workflow and was acceptable to physicians and nurses. However, the low follow-up attendance among high-risk individuals highlights a major implementation limitation, indicating that risk identification alone is insufficient to ensure patient engagement in preventive care. Future development should focus on structured, multichannel engagement strategies and more convenient follow-up models.

## Introduction

Cardiovascular disease (CVD) is a leading cause of mortality among older adults in China, prompting large-scale screening efforts. The China National Basic Public Health Service program provides free annual health checkups for all residents aged ≥65 years, covering approximately 173 million people (12% of the population) [1]. These checkups successfully identify individuals with CVD risk factors or elevated estimated risk. However, postcheckup management of cardiovascular risk remains grossly inadequate. In practice, many high-risk patients do not receive or adhere to recommended follow-up interventions such as lifestyle modification, medication initiation, or specialist referral [2]. This gap is driven by multiple factors: fragmentation of care between checkup providers and primary care or specialty clinics, limited health care resources for follow-up, and poor patient engagement and health literacy [2,3]. Consequently, the benefits of early risk detection are often lost, and preventable CVD events continue to occur.

Digital health interventions offer a promising approach to bridge this implementation gap in CVD prevention [4,5]. A tailored, technology-based system can facilitate continuity of care by automatically identifying high-risk individuals during checkups and actively connecting them with personalized risk management resources [4]. In China, where smartphone use and telehealth are increasingly common even among older adults, a digital tool embedded in the checkup process could overcome some barriers of traditional follow-up (eg, limited clinic time and geographic distance) and reinforce patient engagement outside of hospital visits.

In 2023, we launched a CardioCare program, which is a comprehensive digital CVD risk assessment and management system integrated into health checkup centers. By providing risk stratification, targeted education, and ongoing reminders within a unified platform, the CardioCare program aims to improve risk factor control and encourage preventive care actions in those identified at risk. This report focuses on the design, development, and preliminary testing of the CardioCare system.

## Methods

### Study Design and Setting

For the CardioCare program, phase 1 focused on the development of the system, which was conducted at the Health Management Center of the First Affiliated Hospital of Sun Yat-sen University in Guangzhou, China. This center conducts routine health examinations for residents. Phase 1 activities included system design and software development, followed by a pilot implementation with real patients and providers to assess usability and integration into the clinical workflow. The primary goals were to ensure the system’s technical functionality in a real-world setting and to gather preliminary data on user engagement prior to a future randomized trial. This implementation study is reported in accordance with the iCHECK-DH (Guidelines and Checklist for the Reporting on Digital Health Implementations) statement to ensure transparency, completeness, and reproducibility of digital health reporting [6].

### CardioCare System Overview

**Figure 1.**
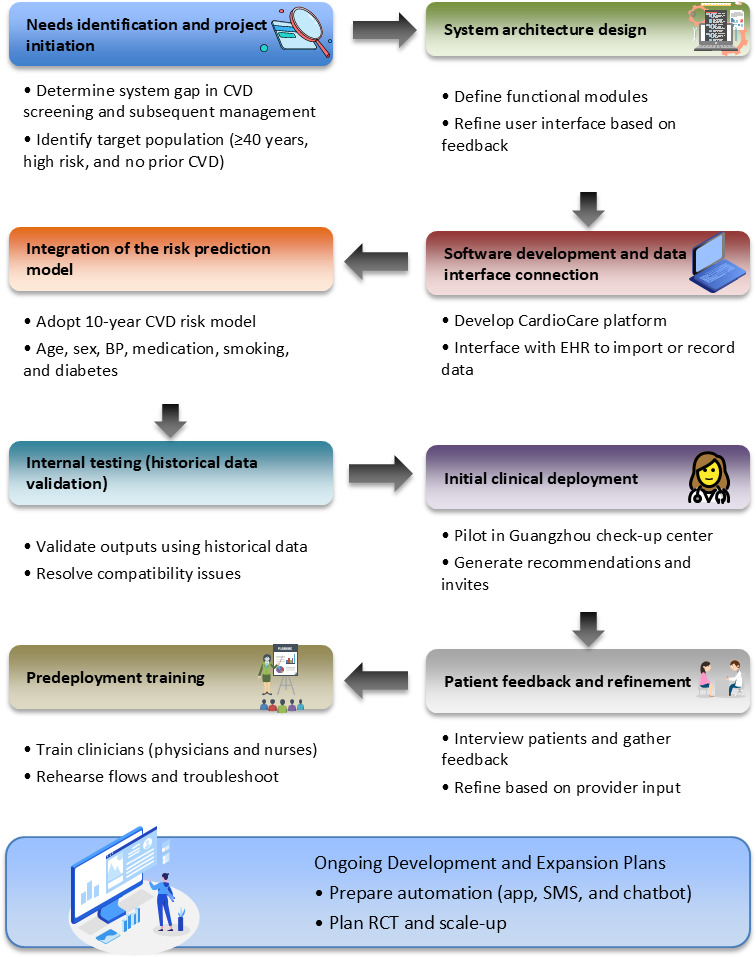
Establishment process of the CardioCare digital risk management system in a health checkup center. BP: blood pressure; CVD: cardiovascular disease; EHR: electronic health record; RCT: randomized controlled trial.

The CardioCare system is a custom digital health software that integrates cardiovascular risk screening with a personalized cardiovascular risk management clinic. Key components of the system include a risk prediction module and personalized health management tools. The CardioCare system incorporates a 10-year CVD risk prediction model based on the Guangzhou Biobank Cohort Study (GBCS) [7]. This validated model uses 6 patient variables (age, sex, systolic blood pressure, use of antihypertensive medication, smoking status, and diabetes status) to estimate the 10-year risk of major cardiovascular events [8]. The model was chosen for its demonstrated predictive performance in a contemporary Chinese population and its practicality (not requiring laboratory cholesterol measurements). During checkups, relevant data are obtained and input into the CardioCare system, either by importing from the electronic health record or by manual entry, after which the system automatically calculates the 10-year CVD risk score (Figure 1). To enhance transparency and replicability, Table 1 summarizes the operational data flow of the CardioCare system, including data inputs (automated vs manual), processing steps, and output delivery mechanisms. Illustrative examples of key system outputs are provided in Multimedia Appendix 1.

**Table 1.** Operational data flow of the CardioCare system.

Stage and data element	Source	Entry mode	Processing	Output
1. Data capture				
Demographics (age and sex)	EHR^a^	Automatic import	Stored on a secure server	Used in risk calculation
Blood pressure	Measured at clinic	Manual confirmation	Integrated with EHR	Used in risk calculation
Smoking status	Checkup questionnaire	Manual entry	Stored	Used in risk calculation
Diabetes status	EHR	Automatic import	Stored	Used in risk calculation
Antihypertensive use	EHR	Automatic import	Stored	Used in risk calculation
2. Risk calculation				
10-year CVD^b^ risk	System algorithm (GBCS^c^ model)	Automated	Server-based computation	Risk score (%) generated
3. Report generation				
Personalized risk report	Auto-generated	Automated	Template+risk profile	PDF report+clinician dashboard
4. Messaging				
Invitation or reminders	System algorithm	Automated	Tailored by risk and engagement	SMS or WeChat message
5. Engagement tracking				
Message opened or clicked	Messaging platform	Automated logging	Stored in database	Engagement dashboard

a EHR: electronic health record.

b CVD: cardiovascular disease.

c GBCS: Guangzhou Biobank Cohort Study.

The CardioCare system is fully integrated into the hospital’s internal health information system and operates within its centralized big data platform. All checkup data, including laboratory test results, clinical history, and medication use, are automatically available to the system without requiring external interface standards. Physical measurements such as blood pressure and anthropometrics may be manually confirmed or entered by clinic staff as needed. The system comprises a web-based front-end accessible to clinicians and a back-end server hosted within the hospital’s secure internal network. Patient data are encrypted during storage, and access is strictly controlled using a role-based permission model. All user activity is logged and routinely audited to ensure data integrity. During the pilot, the system maintained an uptime of more than 99.5%. Minor issues such as brief delays in data loading during peak hours were promptly resolved through server resource optimization. Within 5 working days after the checkup, based on the calculated risk and the individual’s health profile, the system generates tailored recommendations. For example, a patient with hypertension and moderate risk might receive weekly reminders to check blood pressure and daily prompts to take antihypertensive medication. The content and schedule of these messages are customized to each patient’s risk factors and readiness to change.

The system was designed for seamless integration into the existing clinical workflow at the checkup center. Role-based access control is used to ensure data security and patient privacy, meaning that only authorized clinicians (eg, checkup physicians, nurses, and cardiologists involved in follow-up) can access or input data. The user interface was co-designed with input from frontline health care providers to be intuitive and to fit within the time constraints of a busy checkup clinic.

### Preliminary Implementation and Usability Testing

After internal testing with historical patient data to validate system outputs, the CardioCare system was developed in routine clinical encounters over a 6-month pilot period. Eligible participants were adults aged ≥40 years attending routine health checkup at the study site, with an estimated 10-year CVD risk ≥10% and no prior diagnosis of CVD. A 10-year predicted CVD risk of ≥10% was used to define high risk, based on prior validation of the GBCS model [8] and in alignment with risk thresholds used in international guidelines (eg, the World Health Organization and the European Society of Cardiology) for identifying individuals requiring follow-up intervention [9]. These individuals were selected as they represent individuals at elevated risk who are not typically managed in specialist cardiac care settings.

After the checkup visit, the examining physician used the CardioCare system to calculate the patient’s risk and reviewed the result with the patient. Patients above the 10% risk threshold received a personalized CVD risk report and counseling. At the end of the visit, they were formally invited to enroll in a “cardiovascular risk management clinic”—a follow-up and ongoing care program facilitated by the system. Invitations were delivered via system-generated messages outlining the rationale for follow-up and instructions for enrollment. The implementation was governed by a multidisciplinary project team that included epidemiologists, cardiologists, public health experts, clinical managers, and software engineers. Oversight was coordinated by a core leadership group led by the principal investigators, who held monthly project meetings to review progress, resolve operational challenges, and approve any updates to the system.

Health care providers (physicians and nurses) received training on the CardioCare system before the pilot launch. A total of 9 staff members (5 physicians, 2 nurses, and 2 administrative assistants) participated in structured training sessions led by the research team and software developers. Training included hands-on demonstrations, role-based walk-throughs, and case-based troubleshooting scenarios. Throughout the pilot, research staff monitored system performance, including any software stability and data integration, and provided on-site technical support. Key implementation metrics included the number of high-risk individuals identified and their uptake of the cardiovascular risk management clinic invitation. Usability was assessed via a focus group involving 4 physicians, 2 assistants, and 11 participants. Participants were recruited through poster announcements in the clinical workspace and direct invitation from participants at the checkup center. The session was guided by a semistructured discussion outline developed by the research team, which covered topics such as ease of use, perceived usefulness, communication with patients, and barriers to implementation. The discussion was recorded with participants’ consent, and detailed notes were taken by 2 researchers. Audio recordings were transcribed verbatim and analyzed using an inductive thematic approach. Two members of the research team independently reviewed and coded the transcripts to identify recurrent concepts and patterns. Initial codes were generated directly from the data without a predefined framework. Codes were then grouped into broader themes through iterative discussion among the research team. Discrepancies in coding were resolved through consensus. Given the pragmatic implementation focus of the pilot, analysis was conducted using a rapid thematic approach without the use of qualitative analysis software.

Several adaptations were made during deployment based on feedback and real-time performance monitoring. These included optimizing server performance during peak hours to reduce loading delays, simplifying certain data-entry fields to improve workflow efficiency, and updating patient-facing risk reports to enhance clarity and comprehension.

The primary implementation outcomes included feasibility, adoption, acceptability, and engagement. Feasibility referred to the ability of the system to operate reliably in the clinical environment, integrate into routine workflow, and function without major technical issues. Adoption was defined as clinicians’ use of the system for eligible checkup participants following training and deployment. Acceptability encompassed clinicians’ and patients’ perceptions of usability, clarity, and fit within routine care, as explored through provider focus groups and patient feedback. Engagement referred to whether high-risk individuals acted on the follow-up invitation by attending the cardiovascular risk management clinic, with quantitative engagement outcomes reported in the Results section.

All data were stored on secure servers within the hospital’s internal network. Data transmission occurred over encrypted hospital intranet channels, and role-based access control restricted access to authorized personnel only. All access was logged and audited regularly to ensure data integrity and privacy.

### Ethical Considerations

This project was reviewed and approved by the institutional review board of the School of Public Health of Sun Yat-sen University and classified as a quality improvement initiative (institutional review board-2024‐110). All participants provided written informed consent. The study complied with institutional data protection protocols and relevant guidelines for digital health implementation.

## Implementation (Results)

### System Development and Deployment

A multidisciplinary team of epidemiologists, cardiologists, software engineers, and public health experts successfully developed the CardioCare system over a 6-month period. The final system was deployed in the Health Management Center without major technical difficulties. In April 2023, the risk assessment module in the CardioCare system (including the 10-year cardiovascular risk questionnaire and risk scoring system) was officially launched for routine use in the health checkup center. Subsequently, on August 29, 2023, the hospital opened a dedicated postcheckup cardiovascular risk management clinic to offer follow-up services for individuals identified as high risk by the system. During internal testing on retrospective patient records, the automated risk calculation yielded results identical to manual calculations using the published GBCS risk equation, confirming the accuracy of the implementation. No data security or privacy issues arose during this phase; role-based access controls functioned as intended, and all access was logged without breaches.

### Feasibility and Technical Performance

The CardioCare system operated smoothly in the clinical setting. Clinicians were able to complete the risk assessment and review the results within the usual duration of the checkup consultation (no significant delays were reported). The auto-generated patient risk reports and recommendation printouts were successfully produced for all high-risk patients. All clinicians adhered to using the system for every eligible patient, indicating good acceptance. There were a few minor technical issues noted, such as occasional slow loading of patient data during peak clinic hours, but no crashes or loss of data occurred. These issues were resolved by adjusting the server configuration.

### Usability and User Feedback

Feedback from health care providers was largely positive. Clinicians who used the system reported that having an objective risk score improved how they communicated risk to patients, for example, showing a patient that they had “a 20% chance of heart attack or stroke in 10 years” helped prompt discussions about lifestyle changes. However, clinicians noted that the manual process for delivering personalized messages (phone calls and mailing printouts) was labor intensive. Providers suggested automating the patient communication (via mobile app or SMS) as a priority for future development—a feature that was already in our road map (specifically, incorporating an automated chatbot for messaging).

Patient feedback was available only from a small sample (181/2069, 8.7%). Most of these patients reported that the initial risk explanation and the written report were clear and informative. They appreciated the tailored advice. Nevertheless, common themes for why many peers might not follow through included the following: lack of perceived urgency (“If I feel okay, I didn’t think I needed the special heart program”), family or time constraints, and fear or denial about illness—consistent with known barriers to care seeking in this population. A few also mentioned that a phone-based or online format for follow-up might be preferable to in-person clinic visits, especially for those living farther from the hospital.

### Implementation Metrics and Follow-Up Outcomes

Between January 2024 and April 2025, the CardioCare system was used to assess cardiovascular risk among 9148 individuals who submitted data for evaluation during routine health checkups. Of these, 300 were excluded due to a prior diagnosis of CVD, and 8848 individuals underwent CVD risk assessment using the system. Risk stratification results showed that 5179 (58.5%) individuals were classified as low risk, 1600 (18.1%) as moderate risk, and 2069 (23.4%) as high risk (defined as ≥10% 10-year CVD risk). All individuals classified as high risk were invited to attend the cardiovascular risk management clinic for follow-up care. Across the reporting period, a total of 181 high-risk individuals attended the clinic at least once, yielding an overall follow-up rate of 8.7%. Most of these patients (n=152, 83.9%) had only 1 follow-up visit, while a smaller proportion returned for multiple visits. Table 2 summarizes the main implementation outcomes and lessons learned from the pilot deployment of the CardioCare system.

**Table 2.** Summary of implementation outcomes and lessons learned from the CardioCare system pilot.

Implementation domain	Main findings
Feasibility	The CardioCare system was successfully deployed in the health checkup center and integrated with the hospital information system. Risk calculation, report generation, and clinician dashboard functions operated without major technical issues.
Adoption	Clinicians used the system for eligible checkup participants after training. The system was incorporated into routine checkup workflow.
Acceptability	Health care providers reported that the system was user-friendly and useful for communicating CVD^a^ risk. Patients who attended follow-up generally reported that the risk information and advice were clear.
Engagement	Follow-up engagement was low. Among 2069 high-risk individuals invited to the cardiovascular risk management clinic, 181 attended at least once, corresponding to an attendance rate of 8.7%.
Adaptations during implementation	Server performance was optimized to reduce loading delays, selected data-entry fields were simplified, and patient-facing risk reports were revised to improve clarity.
Key barriers	Major barriers included low patient uptake of follow-up care, reliance on one-time invitations, labor-intensive manual patient outreach, and variation in follow-up counseling across providers.
Lessons learned	Technical integration is feasible, but future implementation should include proactive patient engagement, automated multichannel reminders, standardized counseling protocols, and more convenient follow-up options such as remote or community-based care.

a CVD: cardiovascular disease.

## Discussion

We developed and pilot-tested the CardioCare digital health system to enhance CVD prevention following routine health screenings in China. The system’s successful integration into a real-world checkup clinic and positive clinician feedback are encouraging, suggesting that such technology can be embedded in existing health care workflows to support risk assessment and management. However, implementation effectiveness was limited by very low patient engagement with follow-up care. Thus, the main evidence from this pilot supports the feasibility of system integration, while also identifying follow-up uptake as a central barrier that must be addressed before wider implementation.

Despite the promise of the platform, the extremely low uptake of follow-up services among high-risk patients is a critical finding. Only 8.7% (181/2069) of individuals invited to the cardiovascular risk management clinic engaged with the program, even though all had elevated risk and were offered free follow-up. This outcome highlights that identifying risk is not sufficient—the gap between risk detection and patient action remains large. Similar challenges have been observed in other settings where patients, especially older adults, often do not act on preventive care recommendations due to various barriers. Therefore, future implementation should not rely on one-time invitations alone but should incorporate proactive outreach, repeated reminders, simplified appointment scheduling, remote or community-based follow-up options, and strategies to improve risk perception and health literacy.

Among the small proportion of patients who attended follow-up, reported barriers affecting peers included lack of perceived urgency (“If I feel okay, I didn’t think I needed the special heart program”), competing family or time commitments, and fear or denial regarding potential illness. These observations are limited to the views of attendees and may not represent the perspectives of those who did not attend. For the majority of high-risk individuals who did not engage, we did not systematically collect data on reasons for nonattendance. Therefore, explanations such as limited health literacy, low perceived susceptibility, or logistical barriers remain hypotheses rather than empirically confirmed findings. Future iterations should incorporate structured assessment of nonparticipation to better understand engagement barriers.

It is worth noting that our pilot’s follow-up invitation was a one-time effort that relied on patients to proactively attend the clinic, which created a high threshold for participation. In other words, there was no organized, active follow-up mechanism from the system after the initial recommendation—the onus was entirely on the patient to initiate further care. More convenient or compelling modes of engagement might yield better results. For example, home-based or community-based follow-up consultations or leveraging mobile technology for remote management could lower barriers to participation. The CardioCare system is well positioned to evolve in this direction. Future versions of the system will include an automated chatbot and messaging functionality, enabling continuous engagement without requiring frequent in-person visits. This would allow patients to receive advice and monitoring through their mobile devices at home, potentially improving adherence to recommendations. Furthermore, intensified patient engagement strategies will be essential going forward.

Intensified patient engagement, also known as patient empowerment, may include strategies to enhance patient education, incorporate follow-up reminders and navigation, and address practical barriers. First, providing more impactful education at the time of the checkup, such as visual aids to illustrate risk or involving family members in counseling, to ensure patients fully grasp the significance of their results and recommended actions. Improving health literacy and risk perception is fundamental to motivating follow-up. Second, implementing multiple reminder modalities (phone calls, text messages, and WeChat notifications) and offering assistance with scheduling could gently push patients to take the next step. Proactive outreach, rather than a one-time invitation, may convert more high-risk individuals to active participants. However, our initial attempt at using SMS text message alerts to encourage follow-up did not substantially improve the return rate: many patients appeared to either ignore the text messages or not recognize their importance, suggesting that a simple SMS reminder alone was insufficient to overcome the engagement barriers. This indicates that any automated communication must be carefully designed to capture the patient’s attention and convey a sense of urgency. Our finding that a manual follow-up call system is labor intensive supports the need for automated yet personalized reminder systems to sustain patient contact. Finally, aligning follow-up appointments with other convenient services (for instance, same-day multispecialty visits or community clinics closer to patients) and mitigating cost or transport issues (perhaps via coordination with local community health workers) might increase attendance. While these broader health system issues extend beyond the software itself, integration with primary care and community resources is an important consideration for the CardioCare system’s implementation strategy.

Another challenge identified during the pilot was the variability in follow-up care delivery across different providers. The postcheckup cardiovascular clinic was staffed by multiple physicians (specialists taking turns seeing patients), and we observed some inconsistency in how risk information was communicated and how follow-up plans were formulated, depending on which provider saw the patient. In the absence of a unified protocol, each clinician tended to apply their own approach, potentially leading to mixed messages or uneven quality of care. This lack of standardization may reduce the effectiveness of the intervention, as patients could receive different recommendations or levels of follow-up care depending on the clinician. Going forward, establishing clear, standardized protocols for risk communication and follow-up management—and training all participating clinicians in their use—will be crucial to ensure consistency in patient counseling and intervention delivery.

The high acceptance of the CardioCare system by providers is a strength of the project and is expected to support its continued use and refinement. Clinician buy-in supports the consistent use of the system for risk assessment and patient counseling, providing a necessary foundation for improving patient engagement and uptake. However, this study has several limitations. We did not formally analyze reasons for nonparticipation from the majority who declined; thus, our understanding of barriers is based on provider observations and general literature, rather than direct data from nonresponders. Additionally, the pilot was conducted in a single urban hospital center with a particular patient demographic, so the findings (both positive and negative) may not generalize to other regions of China, especially rural areas where resource and literacy barriers could be even greater. Furthermore, on the operational side, not all eligible checkup participants completed the CVD risk questionnaire during their visit, which means a subset of individuals did not receive any risk assessment. This incomplete uptake of the questionnaire likely reduced the program’s reach by leaving some high-risk patients unidentified. Addressing this gap by improving the questionnaire completion rate (through better staff engagement, clearer prompting of patients, or simplifying the questionnaire for older users) will be important to ensure that as many patients as possible are screened and managed. However, the issues identified—such as the burden of manual intervention delivery and poor patient uptake—are likely to be common challenges for any similar digital health initiative aiming to improve postcheckup care.

Several key lessons emerged from this pilot. First, a single-point invitation strategy was insufficient. Reliance on patient-initiated follow-up after one recommendation resulted in very low uptake. Future implementations should incorporate structured, multistep outreach with predefined follow-up triggers. Second, requiring in-person clinic attendance likely limited participation. Hybrid or remote follow-up models should be integrated from the outset to reduce access barriers. Third, variability in clinician communication and management planning highlighted the need for standardized counseling protocols to ensure consistency and fidelity across providers. Fourth, we did not conduct a formal costing analysis in this preliminary implementation report. However, the main resource requirements for implementation included software development and maintenance, integration with the hospital information system, secure server support, staff training, technical support, and staff time for patient outreach and follow-up coordination. During scale-up, manual patient communication and follow-up coordination may become important resource drivers, supporting the need for automated messaging and workflow integration. Finally, incomplete uptake of the risk questionnaire reduced program reach. Workflow integration and system prompts are critical to maximize coverage.

In conclusion, integrating a 10-year CVD risk prediction model with personalized management advice in a digital platform is technically feasible in the context of Chinese health checkup centers. The system operated reliably, fitted into the routine workflow, and was acceptable to clinicians. However, the low follow-up engagement rate among high-risk individuals represents a major implementation limitation and shows that digital risk stratification alone is insufficient to translate screening results into preventive action. Future iterations of the CardioCare program should therefore prioritize structured patient engagement mechanisms, including automated multichannel reminders, standardized counseling protocols, and more convenient follow-up models such as remote or community-based care.

## Supplementary material

10.2196/78942 Multimedia Appendix 1 Illustrative examples of key outputs generated by the CardioCare system, including 10-year cardiovascular disease risk assessment results and personalized risk-management recommendations.

10.2196/78942 Checklist 1 iCHECK-DH implementation report checklist.

## Abbreviations

CVD: cardiovascular disease
GBCS: Guangzhou Biobank Cohort Study
iCHECK-DH: Guidelines and Checklist for the Reporting on Digital Health Implementations
