# Supplementary material for: Design and Preliminary Testing of the CardioCare System in Health Checkup Centers: Implementation Report
Source: JMIR Med Inform. 2026 Jul 13;14:e78942. doi: 10.2196/78942 (PMC13361893; doi:10.2196/78942)
Supplement: Multimedia Appendix 1 [file medinform-v14-e78942-s001.docx]

**Appendix 1 Illustrative Examples of CardioCare System Outputs**

### Example 1. De-identified Personalized Cardiovascular Risk Report

**Delivery channel:** Printed at clinic visit + secure WeChat message
**Timing:** Within 5 working days after check-up

**Patient ID:** CC-2048
**Age:** 63 years
**Sex:** Female
**Systolic Blood Pressure:** 152 mmHg
**Smoking Status:** Non-smoker
**Diabetes:** Yes

**Estimated 10-year CVD Risk:** 21.4% (High Risk Category)

**Interpretation:** Your estimated 10-year risk of developing cardiovascular disease is higher than average for individuals of your age and sex. Elevated blood pressure and diabetes are the primary contributing factors.

**Recommended Actions:**
• Schedule consultation at Cardiovascular Risk Management Clinic within 14 days
• Home blood pressure monitoring (twice weekly)
• Medication review with physician
• Lifestyle modification (dietary salt reduction, moderate exercise ≥150 min/week)

### Example 2. De-identified Invitation Message

**Delivery channel:** WeChat official hospital account
**Timing:** 3 days after check-up

“Dear Ms. L., your recent health examination indicates an elevated 10-year cardiovascular risk (21.4%). We recommend enrollment in the Cardiovascular Risk Management Clinic for personalized follow-up care. Please click the link below to schedule your appointment within the next 14 days.”

### Example 3. Follow-up Reminder (if no appointment scheduled)

**Delivery channel:** SMS
**Timing:** Weekly reminder for up to 4 weeks

“Reminder: Managing blood pressure and diabetes reduces your heart disease risk. Please schedule your Cardiovascular Risk Management Clinic visit. Reply ‘1’ for assistance or call 020-XXXXXXX.”
