# Supplementary material for: Design and Preliminary Testing of the CardioCare System in Health Checkup Centers: Implementation Report
Source: JMIR Med Inform. 2026 Jul 13;14:e78942. doi: 10.2196/78942 (PMC13361893; doi:10.2196/78942)
Supplement: Checklist 1 [file medinform-v14-e78942-s002.docx]

Multimedia Appendix 2. Completed iCHECK-DH Implementation Report Checklist

**Article:** Design and Preliminary Testing of the CardioCare System in Health Checkup Centers: Implementation Report. Locations refer to the proof manuscript pages.

| **Section** | **No.** | **Item** | **Checklist item description** | **Reported in manuscript** |
| --- | --- | --- | --- | --- |
| **TITLE** | **1** | **Title (M)** | Identify the manuscript as an implementation report and describe the implementation in the title and/or keywords. | Reported in the title: 'Design and Preliminary Testing of the CardioCare System in Health Checkup Centers: Implementation Report.' Keywords include 'digital health' and 'risk stratification' (title page, p 1). |
| **ABSTRACT** | **2** | **Abstract (M)** | Summarize the implementation strategy and intervention, key elements, health outcomes, and key performance indicators/outputs. | Abstract includes Background, Objective, Methods, Implementation (Results), and Conclusions. It describes integration of a 10-year CVD risk model, personalized feedback and follow-up invitation, and reports 2069 high-risk individuals invited and 181 (8.7%) attending at least 1 clinic visit (p 1). |
| **INTRODUCTION** | **3** | **Context (M)** | Describe geography, organizations, target populations, implementation context, relevant barriers/facilitators, and the stage of implementation. | The report describes implementation at the Health Management Center of the First Affiliated Hospital of Sun Yat-sen University in Guangzhou, China, among adults attending routine health checkups. It notes fragmented postcheckup care, limited follow-up resources, and poor engagement as barriers. Phase 1 involved system development followed by pilot implementation and evidence generation (Introduction, pp 1-2; Methods: Study Design and Setting, p 2). A specific national digital health strategy was not explicitly described. |
|  | **4** | **Problem statement (M)** | Describe the health care or public health problem, challenge, or deficiency addressed by the implementation. | The implementation addresses the gap between CVD risk detection in routine health checkups and subsequent preventive management. Many high-risk patients do not receive or follow recommended follow-up actions because of fragmented care, limited resources, and low engagement (Introduction, pp 1-2). |
|  | **5** | **Similar interventions (M)** | State whether the implementation was inspired by an existing intervention and describe its added value or differences. | The Introduction cites digital health solutions for CVD prevention and medication adherence. The CardioCare system was developed as a custom hospital-integrated system rather than as an adaptation of a named existing intervention. Its added value is integration of the Guangzhou Biobank Cohort Study 10-year risk model with risk reporting, individualized recommendations, and follow-up referral within the health checkup workflow (Introduction, p 2; Methods: CardioCare System Overview, pp 2-4). |
| **METHODS** | **6** | **Aims and objectives (M)** | Describe the overall aim, objectives, predefined outcomes/key performance indicators, and expected intervention. | The aims were to develop the CardioCare system, assess technical functionality and workflow integration in a real-world checkup setting, and obtain preliminary data on user engagement before a future randomized trial. Implementation outcomes were feasibility, adoption, acceptability, and engagement; key metrics included high-risk individuals identified and uptake of the clinic invitation (Abstract, p 1; Methods, pp 2 and 5). |
|  | **7** | **Blueprint summary (M)** | Describe the intervention design, its key features, and the implementation strategy and roadmap. | Figure 1 presents the roadmap from needs identification, system architecture and risk-model integration through development, internal testing, initial deployment, training, feedback/refinement, and planned expansion. The system overview and operational data flow are described in text and Table 1 (Figure 1, p 3; Methods: CardioCare System Overview, pp 2-4). |
|  | **8** | **Technical design (M)** | Explain the rationale for the tool; describe its functionality, architecture, technology, license/ownership, and relevant documentation or links. | CardioCare is a custom digital health software system that integrates a 10-year CVD risk algorithm with personalized management tools. It uses a web-based front end for clinicians and a back-end server within the hospital's secure internal network; relevant data are automatically imported from the hospital system or manually confirmed/entered as needed. The paper describes functionality and architecture but does not provide public code, a public application link, license details, or intellectual-property ownership (Methods: CardioCare System Overview, pp 2-4). |
|  | **9** | **Target (M)** | Describe the implementation sites, staff/resources, population targeted, and eligibility criteria. | The target site was the Health Management Center at the First Affiliated Hospital of Sun Yat-sen University. Eligible participants were adults aged >=40 years attending routine health checkups, with estimated 10-year CVD risk >=10% and no prior diagnosis of CVD. Clinician users included checkup physicians, nurses, cardiologists involved in follow-up, and administrative assistants (Methods: Preliminary Implementation and Usability Testing, pp 4-5). |
|  | **10** | **Data (M)** | Describe data governance across the life cycle, data protection, consent, cybersecurity, hosting, access, and applicable legal framework. | The report describes collection from electronic health records, clinic measurements, and questionnaires; server-based risk computation; secure storage within the hospital internal network; encrypted intranet transmission; role-based access; logging and auditing; and written informed consent. Data are not publicly available because of privacy considerations and are available from the corresponding author upon reasonable request. Formal data ownership, patient data-access rights, and specific legal-framework details are not explicitly reported (Methods, pp 4-5; Ethical Considerations, p 5; Data Availability, p 8). |
|  | **11** | **Interoperability (M)** | Describe interfaces with other systems and the standards used, including rationale for their selection. | The system is integrated with the hospital's internal health information system and centralized big-data platform. Data from electronic health records are automatically available; physical measurements can be manually confirmed. The manuscript states that no external interface standards were required in this internal environment; named interoperability standards are not reported (Methods: CardioCare System Overview, pp 2-4; Table 1, pp 3-4). |
|  | **12** | **Participating entities (M)** | Describe implementing organizations, partners, government involvement, funders, and ownership of the final product/intellectual property. | Implementation occurred at the First Affiliated Hospital of Sun Yat-sen University, with a multidisciplinary team of epidemiologists, cardiologists, public health experts, clinical managers, software engineers, physicians, nurses, and administrative staff. Oversight was led by the principal investigators through monthly project meetings. Funders are listed in the Funding statement. Government involvement and final-product/IP ownership are not explicitly reported (Methods, pp 4-5; Funding, p 8). |
|  | **13** | **Budget planning (M)** | Describe the planned implementation budget and, when possible, actual costs and their time period. | A formal costing analysis and itemized planned/actual budget were not conducted for this preliminary implementation report. The manuscript identifies the main resource requirements: software development and maintenance, hospital-system integration, secure server support, staff training, technical support, and staff time for outreach and follow-up coordination (Discussion, p 8; Funding, p 8). |
|  | **14** | **Sustainability (M)** | Describe the business/sustainability model, long-term plans, exit strategy, and potential institutionalization. | The system was embedded in routine health checkup workflow within the hospital and continued to operate during the reporting period. Future sustainability and scale-up plans include automated multichannel messaging/chatbot functions, standardized counseling, remote or community-based follow-up, workflow prompts, and a future randomized trial. A formal business model, exit strategy, and cost-effectiveness assessment are not reported (Figure 1, p 3; Discussion, pp 7-8). |
| **RESULTS** | **15** | **Coverage (M)** | Describe the geographic level and relative scale of implementation, including the proportion of the eligible population reached when available. | Coverage was local: one urban hospital health checkup center in Guangzhou. Between January 2024 and April 2025, 9148 individuals submitted data for evaluation, 300 were excluded for prior CVD, and 8848 underwent CVD risk assessment. The denominator for all potentially eligible health checkup attendees was not reported (Results: Implementation Metrics and Follow-Up Outcomes, p 6). |
|  | **16** | **Outcomes (M)** | Report primary and other implementation outcomes using the predefined outcome measures. | Feasibility: successful deployment, integration, risk calculation, report generation, and dashboard operation without major technical issues. Adoption: clinicians used the system for eligible participants. Acceptability: positive provider feedback and favorable feedback from attendees. Engagement: 181 of 2069 high-risk invitees attended at least once (8.7%); 152/181 (83.9%) had only 1 visit. Adaptations and barriers are summarized in Table 2 (Results, pp 5-6; Table 2, p 6). |
|  | **17** | **Lessons learned (M)** | Describe success factors, implementation challenges, operational costs/resource needs, and recommendations for improvement. | Success factors included integration with routine workflow, clinician training and buy-in, secure internal hosting, and multidisciplinary oversight. Challenges included low uptake, reliance on one-time invitations, labor-intensive manual outreach, loading delays at peak hours, incomplete questionnaire uptake, and variation in follow-up counseling. The report recommends proactive multistep outreach, automated multichannel reminders, standardized counseling protocols, improved questionnaire completion, and remote or community-based follow-up (Table 2, p 6; Discussion, pp 6-8). |
|  | **18** | **Unintended consequences (NM)** | Describe unintended positive or negative consequences, harms, or adverse effects, if any. | No unintended clinical harms or data-security/privacy breaches were reported. The system had no crashes or data loss during the pilot. Low follow-up uptake and labor-intensive manual communication were identified as implementation limitations rather than formal unintended-consequence outcomes (Results: System Development and Deployment and Feasibility and Technical Performance, p 5; Discussion, pp 6-8). |
| **DISCUSSION** | **19** | **Conclusion (M)** | Summarize conclusions and future implications. | The report concludes that digital CVD risk stratification and personalized management advice can be technically integrated into Chinese health checkup centers and accepted by clinicians. It emphasizes that risk identification alone did not generate adequate follow-up engagement and that future iterations should prioritize structured engagement, automated reminders, standardized counseling, and convenient follow-up models (Conclusion, p 8). |
| **GENERAL** | **20** | **General (NM)** | Include, where applicable, regulatory/ethical approval, study registration/protocol, data availability, and conflicts of interest. | Ethical approval was obtained from the institutional review board of the School of Public Health of Sun Yat-sen University (IRB-2024-110); participants provided written informed consent. Funding, data availability, and conflicts of interest are reported. Trial registration/protocol availability is not stated; this report describes a pilot implementation before a future randomized trial (Ethical Considerations, p 5; Funding, Data Availability, and Conflicts of Interest, p 8). |

Abbreviations: CVD, cardiovascular disease; EHR, electronic health record; GBCS, Guangzhou Biobank Cohort Study; iCHECK-DH, Guidelines and Checklist for the Reporting on Digital Health Implementations; IRB, institutional review board.
